# Novel Biomarkers in Heart Failure: New Insight in Pathophysiology and Clinical Perspective

**DOI:** 10.3390/jcm10132771

**Published:** 2021-06-24

**Authors:** Luigi Marzio Biasucci, Alessandro Maino, Maria Chiara Grimaldi, Luigi Cappannoli, Nadia Aspromonte

**Affiliations:** 1 Department of Cardiovascular and Thoracic Sciences, Fondazione Policlinico Universitario A. Gemelli, IRCCS, 00168 Rome, Italy; alessandro.maino01@icatt.it (A.M.); mariachiara.grimaldi@unicatt.it (M.C.G.); luigi.cappannoli@gmail.com (L.C.); 2 Catholic University of the Sacred Heart, 00168 Rome, Italy

**Keywords:** heart failure, biomarker, fibrosis, inflammation, noncoding RNA

## Abstract

Heart failure (HF) is a complex clinical syndrome with a huge social burden in terms of cost, morbidity, and mortality. Brain natriuretic peptide (BNP) appears to be the gold standard in supporting the daily clinical management of patients with HF. Novel biomarkers may supplement BNP to improve the understanding of this complex disease process and, possibly, to personalize care for the different phenotypes, in order to ameliorate prognosis. In this review, we will examine some of the most promising novel biomarkers in HF. Inflammation plays a pivotal role in the genesis and progression of HF and, therefore, several candidate molecules have been investigated in recent years for diagnosis, prognosis, and therapy monitoring. Noncoding RNAs are attractive as biomarkers and their potential clinical applications may be feasible in the era of personalized medicine. Given the complex pathophysiology of HF, it is reasonable to expect that the future of biomarkers lies in the application of precision medicine, through wider testing panels and “omics” technologies, to further improve HF care delivery.

## 1. Introduction

Heart failure (HF) has emerged as a growing and significant public health issue due to its epidemic prevalence, to the high rate of morbidity—requiring hospitalization and intensive care—and mortality; approximately 17.9 million people each year die for cardiovascular disease and 9.6% is attributed to heart failure [1].

Thus, the research literature on biomarkers has grown steadily to ensure a timely diagnosis of HF, to inform prognosis, and improve treatment, recently triggered by insights into the pathophysiology of cardiac dysfunction and a greater understanding of the contributing molecular mechanisms.

The pathophysiology of HF is a complex interplay of genetic and multiple molecular mechanisms (from inflammation to hormonal pathways) performing in the composite network of the cardiovascular system environment. Novel and various biomarkers, encompassing enzymes, hormones and biologic substances, are becoming increasingly important for medical everyday praxis since their easy quantification of reliable information significant for diagnostic and prognostic establishment.

Assessment of the clinical potential of a novel biomarker may be structured around three criteria [2,3]. Precise and replicated assessment must be cost-effective and promptly accessible; it must add potential and significant medical information that is not already available from a conscientious clinical evaluation; and, finally, the variety of possible clinical applications of its measurement may enhance the care of patients [2,3].

All these strict criteria are fulfilled by just a few biomarkers, but new different biomarkers may be able to add new pathophysiological insight elucidating clinical scenarios and to guide the precise treatment in HF patients. 

## 2. Novel Biomarkers: Inflammation and Fibrosis

### 2.1. sST2

The source of tumorigenicity 2 (ST2) are receptors for the inflammatory cytokine interleukin (IL)-33 and they are explicated in two forms: membrane-bound and soluble. They belong to the IL-1 cytokine receptor superfamily and their bond with IL-33 indicates to cardiomyocytes and local immune cells the presence of tissue injury in response to myocardial suffering. In experimental models, the overexpression of the IL-33/ST2 system was demonstrated to decrease myocardial fibrosis, apoptosis and to suppress cardiac hypertrophy with the global result of ameliorating heart function [4].

In patients with HF, the principal provenance of sST2 has not been exhaustively certified, but extracardiac production seems predominant [5,6]. The main site of sST2 synthesis in HF is thought to be the lungs [7].

#### 2.1.1. sST2 for HF Diagnosis

The concentrations of sST2 are usually greater in patients with HF, but this biomarker is not helpful for the diagnosis of HF because of its lack of high specificity. In fact, unlike classic cardiac biomarkers such as natriuretic peptides, sST2 levels could be elevated in HF patients as well as healthy subjects or patients with inflammatory diseases such as pneumonia and BPCO [8]. Therefore, sST2 assessment is not useful to distinguish the etiology of dyspnea, in particular between cardiac and extracardiac causes [9].

This biomarker has been studied also in HFpEF and its significance has not emerged clearly. In a population of hypertensive patients, a study by Wang et al. showed sST2 as relatively more efficacious than natriuretic peptide in the recognition of HFpEF, whilst sST2 concentrations were more elevated in HFrEF than in HFpEF patients [10]. 

#### 2.1.2. sST2 and HF Prognosis

In acute HF, the main role of sST2 seems to be prognostic because of its great ability to corroborate risk stratification, in particular for fatal endpoints. Aimo et al. demonstrated, in a large meta-analysis, that sST2 concentrations, both on admission and at discharge, were strongly predictive of all-cause and cardiovascular mortality. Therefore, sST2 plasma levels at discharge seem to predict hospital readmission for HF [11].

Studies have also shown that serial sST2 measurement may yield further prognostic details. In a study of 150 patients hospitalized for acute decompensated HF and undergoing a daily hematochemical parameters assessment, Boisot and colleagues showed that percent change in ST2 can significantly predict short-term mortality [12]. Another study showed that, among patients admitted with acute HF, the subjects with high sST2 levels on admission, and persistently elevated during the hospitalization, bore the highest long-term mortality [13].

In chronic HF, several studies demonstrated a significant prognostic value of sST2, independently from classical biomarkers and other prognostic variables [14]. In patients with end-stage HF, high levels of sST2 were correlated with greater risk for HF therapy (pharmacological or device-assisted) default [15].

Pascual-Figal et al. explored, in their case-control study, the prognostic value of sST2 in patients with mild-to-moderate HF with LV systolic dysfunction: sST2 levels could significantly predict sudden cardiac death [16].

### 2.2. Galectin 3

Galectins are part of a group of beta-galactoside binding lectins, mainly expressed in activated macrophages. They are involved in several cellular and tissutal processes, encompassing cell growth and death, inflammation and tissue fibrosis [17,18].

Galectins are produced in the cytoplasm and explicate their role in the nuclear as well as extranuclear sections [18].

Galectin-3 (Gal-3) can switch silent fibroblasts to active myofibroblasts, which is the hallmark event in tissue fibrosis [19]. Therefore, its interaction with cardiomyocytes prompts collagen I production. These described processes are key events in tissue fibrosis [20,21]. 

In a clinical point of view, several clinical studies of HF indicate that Gal-3 is a biomarker not organ-specific but specific for individual pathogenesis, in particular inflammation or fibrosis. Gal-3 does not demonstrate a great diagnostic value and the benefit of its use is principally for prognostic information. Clinically, the serum Gal-3 levels in patients with HF are significantly increased [22,23,24], and the continuously increasing Gal-3 levels are often associated with a greater risk of adverse cardiovascular events [25,26]. In two large, community-based cohort studies—the Rancho Bernardo Study and the report by De Boer et al.—high levels of Gal-3 were significantly correlated with all causes and cardiovascular mortality [27,28]. In acute decompensated HF, Gal-3 was demonstrated to yield prognostic information used with classic biomarkers [29,30]. 

The serial assessment of Gal-3 has also been shown to be of prognostic value when measured serially in an ambulatory setting. In chronic systolic HF, Gal-3 can independently predict all-cause mortality [22,31,32].

### 2.3. Myocardial Fibrosis and Collagen Synthesis Markers (PICP, PIIINP, CITP)

Myocardial fibrosis plays a pivotal role in structural myocardial remodelling in HF and, therefore, several candidate molecules have been investigated in recent years as possible myocardial fibrosis biomarkers, both for prognosis, diagnosis and response to therapy [33], with only a few of them confirming their role. Myocardial fibrosis occurs when the production of type I and type II collagen exceeds its degradation and this can lead to two types of fibrosis: a macroscopic replacement fibrosis, which is typical of the post-ischemic scar, and a microscopic, diffuse, reactive fibrosis, involving the interstitial and perivascular space. This form is typical of a chronic condition such as hypertension, aortic stenosis, cardiomyopathies, and other causes of chronic HF and it happens when collagen type I fibers exceed type III fibers [34].

The most direct way to evaluate myocardial fibrosis, both qualitatively and quantitatively, has traditionally been myocardial biopsy and, therefore, several candidate biomarkers of myocardial fibrosis have been correlated with the evidence of fibrosis at biopsy. Interstitial fibrosis has been related with LV systolic and diastolic dysfunction, arrhythmias, and sudden cardiac death risk and it was revealed as a possible predictor of the response to therapy in HF [35].

Among the candidates circulating biomarkers, three of them have proved to correlate with histological myocardial fibrosis: serum carboxy-terminal propeptide of procollagen type I (PICP), serum amino-terminal propeptide of procollagen type III (PIIINP), and serum collagen type I telopeptide (CITP) [36].

PICP is formed during the extracellular conversion of type I procollagen into type I collagen and its concentrations increase in patients suffering from HF. Moreover, they correlate with the degree of dysfunction in patients presenting with reduction of ejection fraction (HFrEF) [37], and with mortality in both HF with preserved EF and HFrEF [38]. PICP has also been related to the occurrence of ventricular arrhythmias in advanced HF [39] and its concentration change in response to treatment with drugs such as loop diuretics and mineralocorticoid receptor antagonists.

PIIINP originates from the extracellular conversion of type III procollagen to type III collagen and its concentration correlates with the amount of myocardial tissue replaced by collagen III fibers, the so-called myocardial collagen III volume fraction (CIIIVF), measured with specific imaging techniques [40]. Moreover, HF treatment with spironolactone has been associated with the concomitant reduction of the CVF extension and PIIIN concentration. Lately, serum PIIINP correlates with outcomes and severity of HF [38].

CITP and, more specifically the CITP to serum matrix metalloproteinase (MMP)-1 ratio, as expression of the collagen resistance to MMP degradation, independently correlates with the risk of hospitalization and could therefore help to identify those patients at highest risk [40].

### 2.4. ET-1 and MPO

Endothelin-1 (ET-1) is a peptide involved in renal function regulation and, more generally, vascular tone, affecting urine production, but also water homeostasis [41,42]. It is also a potent vasoconstrictor both for pulmonary and peripheral circulation. The endothelin system is therefore involved in water/sodium homeostasis and extracellular water expansion, and consequent congestion, which characterizes all stages of HF. It has already proved to be involved in the pathogenesis of diseases such as pulmonary hypertension and HF. Elevated blood levels of ET-1 were shown to be linked to the outcomes and the severity of the HF disease [43,44,45]. Compared with healthy subjects, patients with more severe forms of chronic HF presented elevated ET-1 plasma levels, and this may be of pathophysiological significance [42]. ET-1 in HF patients plays a role in neurohormonal activation, hemodynamic deterioration, and cardiovascular remodelling. In a study that enrolled more than 2300 HF patients, ET-1 concentration was measured at baseline and related to outcomes at a 23-month follow-up. Baseline concentration of ET-1 was proportional to the severity of disease (in terms of NYHA class and LVEF) and it was revealed to be an independent outcome predictor of all-cause morbidity and mortality [44]. More recently, ET-1 role was also investigated in acute HF patients, and it was demonstrated that its concentration correlates with clinical signs of congestion (fluid overload), low natriuresis (i.e., urine sodium excretion), and, finally, that levels of ET-1 are also a significant prognostic factor of one-year mortality due to acute HF [46].

Myeloperoxidase (MPO) is an enzyme produced by leukocytes during the inflammatory response to several stimuli, causing the formation of reactive species responsible for tissue oxidative damage and with therefore a pivotal role in the pathogenesis of atherosclerosis, plaque vulnerability, and ventricular remodelling [47]. Several studies demonstrated that MPO plasma concentrations are associated with the prevalence of chronic HF [48] and that can also predict the risk of development of HF [49]. Finally, MPO plasma levels also correlate with the severity of HF by echocardiographic assessment and with long-term outcome [50]. On the other hand, less evidence emerged for the usefulness of MPO in acute HF settings, even if a study suggested it as a possible predictor of one-year mortality [51].

ET-1 and MPO are also, by now, the only two (inflammatory) biomarkers with a proven role in predicting cardiotoxicity from cancer drugs [52,53]. ET-1 was the first indagated and it was proved that its concentration increased after treatment with doxorubicin together with serum lactate dehydrogenase and creatine phosphokinase and that this correlated with the development of doxorubicin-induced cardiomyopathy [52]. Later, Ky et al. demonstrated that only MPO levels, among other candidate biomarkers, were an important predictor of cardiotoxicity after 15 months from different regimens of cancer treatment [53].

### 2.5. Growth Differentiation Factor-15 (GDF-15)

Growth-differentiation factor-15 (GDF-15) is a cytokine with a yet unclear function, but a quite ubiquitous distribution in human tissues and whose increase is associated with cardiac, pulmonary, and renal diseases. Evidence suggests that its plasma concentrations increase in case of different forms of cardiac stress, such as pressure overload, and that, even if a protective role has been hypothesized for this molecule, its increase correlates with enhanced mortality [54].

Different studies demonstrated that high concentrations of GDF-15 correlate with all-cause mortality both in HF, acute coronary syndromes and even in healthy people [55,56]. GDF-15, along with other biomarkers, has emerged to be a strong predictor of all-cause mortality but also of cardiovascular death and myocardial reinfarction [57].

Furthermore, GDF-15 has also been investigated in predicting LV remodelling and systolic dysfunction, and it was demonstrated to be associated to LV diastolic volume increase and EF decrease at 12 months follow-up of patients who suffered from myocardial infarction [58].

Lastly, GDF-15 has been tested also in HFpEF and diastolic dysfunction, with evidence to correlate with structural and functional indices and the degree of diastolic dysfunction [59].

### 2.6. Serum Free Light Chains (sFLC)

A strong interest in innate and adaptive immune response has progressively arisen in recent years as a pivotal mechanism of atherogenesis and endothelial dysfunction and therefore of both ischemic heart disease and HF. sFLC, produced by B lymphocytes, are present in low concentrations in many biological fluids, such as urine, synovial fluid, and serum [60]. An increase in serum levels may be the result of various clinical situations such as inflammatory diseases, renal failure, depression, plasma cell dyscrasia, and are conventionally associated with monoclonal gammopathies. However, a polyclonal increase, both for kappa (κ) and lambda (λ) chains, may occur in autoimmune and other chronic inflammatory conditions [61]. In this context, the role of sFLC in determining cardiovascular disease, in particular ischemic heart disease and HF, has been investigated by several studies. Bellary et al. suggested their possible role for the stratification of cardiovascular risk in patients with or without type 2 diabetes mellitus [62]; Shantsila et al. proposed sFLC as a predictive marker of mortality in patients with acute HF and acute coronary syndromes [63,64]. Serum FLC increase has already been proved to correlate with an increased risk of mortality. In their study, Dispenzieri et al. analyzed mortality and death causes in more than 15,000 patients, proving that a non-clonal increase of sFLC was a strong mortality predictor also for patients without plasma cell disorders [65]. Burmeister et al. further investigated the role of sFLC in different chronic and acute diseases, comparing their concentration to that of high sensitive reactive protein (hs-CRP), a well-known marker of flogosis and cardiovascular diseases. They found a weak correlation between polyclonal sFLC levels and hs-CRP, thus suggesting different, and possibly complementary, mechanisms in determining such diseases and inflammatory status [66]. More recently, Basile et al. documented high values of sFLC in patients with ischemic heart disease, in particular in those with NSTEMI presentation, and in those with type 2 diabetes mellitus. They also confirmed the previously mentioned lack of correlation between sFLC and hs-CRP concentrations and, finally, they also reported that a high κ/λ light chains ratio at hospitalization correlated with a 12-month LVEF improvement, with a yet unclear pathophysiological mechanism, but suggesting a possible role in predicting post-ischemic HF [67].

In conclusion, sFLC, produced by the B-lymphocytes and expression of adaptive immunity, could have a pivotal role in the pathogenesis of cardiovascular diseases and could therefore represent a novel risk biomarker in HF.

**Figure 1:**
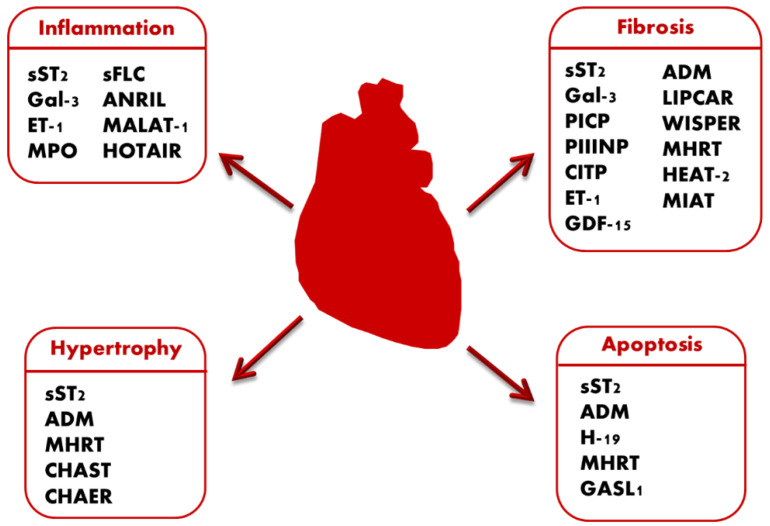
Pathophysiological pathways in heart failure and involved biomarkers. sST2 = soluble source of tumorogenesis-2; Gal-3 = galectin-3; PICP = serum carboxy-terminal propeptide of procollagen type I; PIIINP = serum amino-terminal propeptide of procollagen type III; CITP = serum collagen type I telopeptide; ET-1 = endothelin-1; MPO= Myeloperoxidase; GDF15 = Growth-differentiation factor-15; s-FLC = serum free light chain; ADM = adrenomedullin.

A summary of the biomarkers discussed in this section can be found in Table 1 and Figure 1.

## 3. Neurohumoral Biomarkers

### 3.1. Adrenomedullin (ADM)

Adrenomedullin (ADM) is a peptide hormone involved in cardiovascular and renal functions, fluids and sodium homeostasis, and with inotrope effects on the heart. As other natriuretic peptides (brain natriuretic peptide (BNP), atrial natriuretic peptide (ANP), and C-natriuretic peptide), ADM is involved in vascular tone control, acting as a strong vasodilator, and preserving endothelial integrity. In vitro studies and in murine models, ADM showed a protective effect on the heart reducing fibrosis and cardiomyocyte and endothelial apoptosis, induced by oxidative stress [68]. Volume overload is one of the main triggers for ADM secretion; thus, it may have a role in HF as a biomarker of tissue congestion [69].

Previous studies demonstrated that ADM acts as an independent predictor for adverse prognosis in HF patients. In a study involving 117 ambulatory patients, ADM plasmatic levels were higher in HF patients than in the control group. This study confirmed ADM as a potent and significant predictor of outcome in HF. Moreover, there were no differences in plasmatic levels of ADM according to HF etiology (ischemic or non-ischemic HF), but higher levels of ADM were associated with decompensated and severe stages of HF [70] (Table 1 and Figure 1).

### 3.2. Copeptin

The hypothalamic hormone arginine vasopressin (AVP), also known as anti-diuretic hormone, is involved in fluid and sodium homeostasis and participates to control plasma osmolality and arterial pressure. In HF, AVP secretion is increased in response to the hypovolemic state and the low cardiac output. AVP has a short half-life, while its C-terminal inactive fragment, copeptin, is a more stable and easily detectable form. In a multicenter study involving 268 HF patients, higher levels of copeptin were significantly linked to re-hospitalization and death. Additionally, in this study, copeptin resulted in being significantly superior to BNP and NT-proBNP about its predictive role [71]. Similarly, in a metanalysis comprising 4473 acute and chronic HF, copeptin resulted as a strong predictor of all-cause mortality, comparable with NT-proBNP [72] (Table 1).

## 4. Novel Biomarkers: Ribonucleic Acids

The Human Genome Project showed that less than 3% of the total transcriptome is coding for proteins. The 97% of the total RNA—not translated into proteins—was deemed as “genetic junk” and supposed to be unfunctional for the cellular environment. Recent findings added growing evidence that previously dismissed “junk DNA” produces RNA molecules with important regulatory functions. Indeed, noncoding RNAs (ncRNAs) provide novel insight into the pathogenesis of the diseases, playing a critical role in genetic modulation. According to nucleotide length, ncRNAs can be divided into (1) small ncRNAs (<200 nucleotides), which include microRNA (miRNA), PIWI-interacting RNA (piRNA), circular RNA (circRNA) and small interfering RNA (siRNA), and (2) long non-coding RNA (lncRNA), greater than 200 nucleotides. Although the role of miRNA has been widely examined in cardiovascular science, little is known about lncRNA.

### Long Noncoding RNA and HF

The complex molecular structure of lncRNAs reflects their multiple and intricate functions. They are master regulators of all biological functions and drive gene expression through transcriptional and post-transcriptional mechanisms. LncRNAs can perform as a molecular guide, scaffold, decoy, sponge for microRNA, folding and methylation machinery, gene silencing and activation. Unlike protein-coding genes and miRNA, lncRNAs are different between species and are highly tissue-specific [73,74,75].

Despite the abundance of exonucleases in blood circulation, different types of circulating RNAs have been found intact. Both coding and ncRNAs are protected from degradation packed in vesicles (exosomes and microvesicles), bound to lipoproteins and proteins.

LncRNAs can be detected in body fluids, such as plasma, and manifest variability upon different stages of the diseases. The long-term stability of lncRNAs makes them a possible novel class of non-invasive prognostic and diagnostic biomarkers.

Previous studies elucidated that lncRNAs are masters of epigenetic regulation during heart development [76], cardiac hypertrophy [77], cardiac fibrosis [78], and HF [79]. 

Different studies demonstrated that specific lncRNAs can be re-expressed in mouse and human models of HF, guiding the re-expression of the fetal genes [80].

The first lncRNA identified as a potential biomarker of HF is LIPCAR (long intergenic noncoding RNA predicting cardiac remodelling), a mitochondria-derived lncRNA found in the plasma of patients with maladaptive LV remodelling after myocardial infarction (MI). Plasma levels of LIPCAR were validated in a prospective multicenter study involving 246 patients with MI. LIPCAR was associated with an increased risk of developing HF and adverse prognosis [81]. LIPCAR was supposed to modulate mitochondrial function. Moreover, Zhang et al. found high plasma levels of LIPCAR and the imprinted lncRNA H19 in a prospective study including 300 patients with coronary artery disease and concomitant HF [82].

The cardiac fibroblast–enriched lncRNA WISPER (Wisp2 super-enhancer–associated RNA), is a critical regulator of fibroblast proliferation, migration, and survival. WISPER overexpression increased fibrosis in a murine heart model of MI and in human cardiac biopsies from patients with aortic stenosis. In a mouse model, WISPER knockdown significantly decreased myocardial fibrosis and improved myocardial function. Acting as a super-enhancer, WISPER regulates specific genes involved in fibrosis (as COL1A1, COL3A1, FN1, and aSMA in cardiac fibroblasts). In patients with aortic stenosis, WISPER expression was correlated with the development of severe fibrosis, supporting the translational perspectives of this lncRNA into clinical scenarios as a biomarker and its potential role as an antifibrotic therapeutic target [83].

LncRNAs CHAST and CHAER are also involved in maladaptive cardiac remodelling through epigenetic reprogramming and induction of hypertrophic genes [84,85].

In a murine model, silencing the expression of ANRIL (antisense lncRNA in the INK4 locus) improved the maladaptive myocardial remodelling and reduced the expression of inflammatory factors in ischaemic myocardial tissue of diabetic rats, by inhibiting myocardial oxidative stress [86,87].

MALAT1 (metastasis-associated lung adenocarcinoma transcript 1) was associated with cardiac fibrosis after MI. Moreover, in a diabetic mouse model, silencing MALAT1 improved LV function by reducing cardiomyocyte apoptosis. MALAT1 may become a therapeutic target for diabetic cardiac dysfunction [88].

Moreover, two circulating lncRNAs, MIAT (myocardial infarction-associated transcript) and SENCR (smooth muscle and endothelial cell-enriched migration/differentiation-associated lncRNA) were independent predictors of LV cardiac remodelling in HFpEF diabetic patients [89].

Yanqing Qi et al., reported that MIAT upregulation increased IL-17 production in cardiomyocytes, acting as a sponge for miR-214 and enhancing inflammation [90].

HEAT2 (heart disease-associated transcript 2) is an immune cell-enriched lncRNA highly expressed in the blood of patients with HFrEF [91]. HEAT2 regulates fibroblast proliferation, adhesion, invasion, and transmigration, possibly modulating histone H3K27me3 [92].

Other recent studies identified on plasma samples the circulating lncRNAs NRON (ncRNA repressor of NFAT) and MHRT (myosin heavy-chain-associated RNA transcript) as further independent predictors for HF [93]. NRON regulates intracellular levels of Ca^2+^ through NFAT, which expression and activity are highly increased in HF [94].

MHRT regulates the chromatin-remodelling molecular machinery, preventing the activation of specific gene targets. In vitro studies on cardiomyocytes, MHRT expression, intensely stimulated by oxidative stress, suppressed cardiomyocyte H_2_O_2_-induced apoptosis. In addition, MHRT demonstrated to protect the heart from hypertrophy and HF [95,96]. 

Greco et al. identified a pool of 14 lncRNA in HF patients on LV biopsies. Among them, a pool of nine lncRNAs (ANRIL, EGOT, H19, HOTAIR, TUSC7, RMRP, RNY5, SOX2-OT, and SRA1) were validated in end-stage HF patients [97].

Moreover, lncRNA GASL1 (growth arrest associated lncRNA 1) expression is downregulated in HF. In a mouse model, GASL1 upregulation improved myocardial function by the inactivation of pro-apoptotic factor TGF-b [98].

The lncRNAs previously discussed are listed and summarized in Table 2 and Figure 1.

In sum, these data suggest that lncRNAs may perform critical roles in HF as masters of regulatory commitment and function for different types of cells. Novel therapeutic opportunities might arise from lncRNAs, as modifying the gene expression program leading to HF.

## 5. Conclusions

Novel biomarkers in HF may support the traditional ones—routinely used—by improving diagnosis and prognosis and so enhancing the care of patients. There is a growing interest in the multi-marker approaches because of their benefit over single biomarkers to increase the diagnostic accuracy and to improve risk stratification in HF. Otherwise, further research is needed to identify the best biomarker combination for the management of HF therapy. For example, when myocardial fibrosis is present, anti-fibrotic therapy could be effective; in the absence of myocardial fibrosis, anti-inflammatory therapy or inhibition of inducible nitric oxide synthase may be appropriate.

Noncoding RNAs are attractive biomarkers for their potential clinical applications in personalized medicine. The identification of regulatory lncRNA profiles in HF may give a benefit to existing tools and biomarkers, giving molecular snapshots of the different stages of the disease. It may also lead to new preventive measures, diagnostics, and therapies and it may provide additional information to guide clinical decisions.

The regulatory role of lncRNA might be involved in the positive effects of the last drugs introduced in HF therapy landscape—as sacubitril/valsartan and iSGLT2—by improving the cardiomyocyte cellular dynamics, as mitochondrial function, through epigenetic reprogramming.

Unlocking the complex interplay between the different types of RNAs, and how they can affect gene expression and cellular function, will help to fill critical gaps in our knowledge, enabling such translational applications. How to translate research findings into biologically and clinically relevant innovation is the biggest challenge.

## 6. Limitations

In this review, the authors reported a limited selection among all the possible novel and promising biomarkers related to HF. The authors selected few biomarkers involved in inflammation, fibrosis, apoptosis and hypertrophy pathogenic pathways, according to their translational implications. Although all examined biomarkers might be potentially useful in a clinical setting, a small part of them have been validated in clinical studies involving large groups of HF patients. In the precision medicine era, further investigations are required to assess the clinical relevance of existing candidate biomarkers and to identify new ones.

## Figures and Tables

**Table 1:** Clinical role of the novel biomarkers of inflammation and fibrosis in HF.

	Diagnosis	Prognosis Acute HF	Prognosis Chronic HF	Reference
sST2	-	+	++	[9,11,12,14,16]
Galectin-3	-	++	+	[23,25,28]
PICP-PIIINP-CITP	+	n/a	+	[36,37,38,39,40]
ET-1	-	+	+	[43,44,45]
MPO	+	-	+	[48,49,50]
GDF-15	-	-	+	[55,56,58,59]
s-FLC	-	+	+	[62,66,67]
ADM	-	+	++	[68,69,70]
Copeptin	-	+	+	[71,72]

sST2 = soluble source of tumorogenesis-2; PICP = serum carboxy-terminal propeptide of procollagen type I; PIIINP = serum amino-terminal propeptide of procollagen type III; CITP = serum collagen type I telopeptide; ET-1 = endothelin-1; MPO = Myeloperoxidase; GDF15 = Growth-differentiation factor-15; s-FLC = serum free light chain, ADM = adrenomedullin.

**Table 2:** Long noncoding RNA in HF.

lncRNA	Experimental Model	Mechanism of Action	Detection	Reference
LIPCAR	Maladaptive remodelling (human)	Supposed mitochondrial pathways regulation	Plasma	[80,81]
WISPER	Fibrosis and maladaptive remodelling (mouse, human)	Super-enhancer for genes involved in fibrosis	Myocardial biopsy	[83]
CHAST	Cardiac remodelling (TAC in mouse and human AoS)	Regulation of autophagy and hypertrophy	Myocardial biopsy	[84]
CHAER	Cardiac remodelling (TAC in mouse), dilated cardiomyopathy (human)	Epigenetic reprogramming by interacting with polycomb repressor complex 2	Myocardial biopsy	[85]
ANRIL	Inflammation, cardiac remodelling (mouse, human)	NF-kB pathway and epigenetic reprogramming	Myocardial biopsy	[86,87]
MALAT1	Diabetic cardiomyopathy, inflammation (mouse, human)	Epigenetic control of inflammation	Myocardial biopsy	[88,89]
MIAT	Diabetic cardiomyopathy, inflammation (human)	Promoting cardiac fibrosis by IL-17 production	Serum	[89,90]
SENCR	Diabetic cardiomyopathy (human)	Control of smooth muscle cell phenotype	Serum	[89]
HEAT2	Cardiac remodelling and fibrosis (human, mouse)	Histone metilation	Serum	[91,92]
MHRT	Cardiac remodelling, hypertrophy (mouse, human)	Chromatin remodelling by helicase BRG1 inhibition	Plasma	[93,95,96]
NRON	Cardiac remodelling (mouse)	Regulation of NFAT and Calcium pathway	Plasma	[93,94]
GASL1	Cardiac remodelling (mouse)	Cardiomyocyte apoptosis modulation by TGF-b pathway	Plasma	[98]
HOTAIR	Inflammation, apoptosis (human, mouse)	Modulation of NF-κB pathway	Myocardial biopsy	[84,92]
